# Mechatronic automatic control system of electropneumatic manipulator

**DOI:** 10.1038/s41598-024-56672-4

**Published:** 2024-03-23

**Authors:** Olena Nazarova, Volodymyr Osadchyy, Taras Hutsol, Szymon Glowacki, Tomasz Nurek, Vadym Hulevskyi, Iryna Horetska

**Affiliations:** 1 Department of Electric Drive and Automation of Industrial Equipment, Zaporizhzhia Polytechnic National University, Zhukovsky, 64, Zaporizhzhia, 69-063 Ukraine; 2 Department of Mechanics and Agroecosystems Engineering, Polissia National University, Zhytomyr, 10-008 Ukraine; 3 Department of Fundamentals of Engineering and Power Engineering, Institute of Mechanical Engineering, Warsaw University of Life Sciences-SGGW, 02-787 Warsaw, Poland; 4 Department of Biosystem Engineering, Institute of Mechanical Engineering, Warsaw University of Life Sciences-SGGW, 02-787 Warsaw, Poland; 5 Department of Electric Power Engineering and Electrical Technologies, Faculty of Energy and Computer Technology, Dmytro Motornyi Tavria State Agrotechnological University, Melitopol, Ukraine; 6 Innovative Program of Strategic Development of the University, European Social Fund, University of Agriculture in Krakow, 30-149 Kraków, Poland; 7 Odesa State Agrarian University, Odesa, 65-012 Ukraine; 8 Ukrainian University in Europe – Foundation, Balicka 116, 30-149, Krakow, Poland

**Keywords:** Engineering, Materials science

## Abstract

Mechatronic systems of electropneumatic automation are one of the main classes of industrial automation systems. A laboratory stand for the study of the mechatronic system of automatic control of the pneumatic manipulator and a computer model for preliminary experiments on the adjustment of the automatic control system were developed. Manual and software control modes are provided for research of indicators of safety and quality of management in both modes. To implement the software control mode, a microcontroller part of the laboratory stand based on ADuC841 was developed, with the help of which it is possible to simulate a part of a certain technological process, to detect and eliminate faults in the automatic control system. A study of automatic control systems using a traditional relay-contactor control system, based on GrafCet technology and using a virtual controller. The combination of computer modeling of technological processes and physical modeling of executive mechanisms is a kind of digital double that displays its state, parameters and behavior in real time. The use of a laboratory stand in combination with an adequate simulation model reduces the complexity of developing control systems for practical applications, and also contributes to the formation of students' creative component, ability to analyze the results, and make decisions in unusual situations, which will increase their theoretical and practical training. The study of mechatronic systems of pneumatic manipulators will allow to increase their efficiency and productivity, to optimize their speed and accuracy for various applications in production. The interaction of mechatronic systems of pneumatic manipulators with other technologies, such as machine learning, artificial intelligence, IoT is the basis for creating more integrated and intelligent systems.

## Introduction

Mechatronics is a new field of science and technology that provides production processes based on the integrated use of knowledge in the fields of mechanics, electronics, automation and information technologies. When modeling mechatronic systems, an approach in which the general mechatronic system is considered as a combination of separate systems that have common mathematical or physical characteristics is considered promising. Each of the individual parts of the system can then be modeled using a variety of software and hardware tools that are best suited to investigate specific features of that system. However, at the same time, the connection of individual systems with each other must be preserved. Often, the driving force in a mechatronic system is created by an electric or pneumatic drive, or their combinations. These parts are controlled by automatic control systems. To organize the correct interaction of pneumatic and electrical elements with a relay-contactor or microprocessor system, it is necessary to know the technical parameters of these systems and to be able to debug them with the help of tool software^1^. Providing educational laboratories of universities with the most modern laboratory stands and computers, the use of information technologies in the educational process, the development of innovative infrastructure on the basis of the university are the keys to training a successful specialist^2^. The implementation of the best global developments in the system of domestic professional and higher education also occurs during training and participation of students in all-Ukrainian and international "Worldskills" competitions in the field of "Mechatronics"^3,4^.

In Europe and the world, mechatronic automatic control systems are widely used in various industries, including manufacturing, transport, medicine and science. Research on pneumatic manipulators is aimed at the development and improvement of automated systems that use pneumatic elements to perform various tasks. Scientists from Britain and Italy compared two adaptive laws that compensate for the influence of external disturbances on system dynamics^5^. The performance of the controller is demonstrated through simulations and experiments with two different prototypes. Researchers in China are studying how to improve the efficiency and productivity of pneumatic manipulators, optimize their speed and accuracy for various applications in manufacturing^6^. Scientists from the Republic of Korea and Japan have developed a fast, accurate and inexpensive pneumatic actuator with controlled position, which can be applied to a variety of practical positioning applications with various external loads^7^. Research by scientists from Canada and France is aimed at creating more flexible and adaptive pneumatic manipulators that can quickly adapt to changing conditions and tasks^8,9^. Development and improvement of safety systems for pneumatic manipulators, as well as research into the possibilities of their cooperation with people in work environments. This paper^10^ reviews a study of soft pneumatic actuators carried out in Italy, focusing on mechanical design, analytical modeling and possible applications. European companies and research institutions are actively working on the development and implementation of innovative mechatronic solutions to improve the quality and productivity of industrial and technological processes.

The purpose of the work is the development and research of a mechatronic system of automatic control of a pneumatic manipulator, which can be used to conduct preliminary experiments in the development of new and modernization of existing systems.

To achieve the goal, a laboratory stand was developed with the possibility of manual and software control modes; computer model of the mechatronic system of the electropneumatic manipulator; experiments were carried out on this model with a relay-contactor system of automatic control and with the use of a virtual controller.

## Literature review

Modernization of industrial production all over the world takes place with the use of intelligent technologies, advanced automated control systems. The implementation of such technologies requires specialists who have comprehensive knowledge and skills in the field of mechatronics and electrical engineering^11^. Robotics, mechatronics, automatic control systems with an intelligent component play a fundamental role in achieving the Sustainable Development Goals by replacing and supporting human activity, promoting innovation, improving remote access, and improving monitoring^12^. Grasping robots that repeat the movement of a human hand open up opportunities for people without limbs or as an auxiliary function for people who have difficulty controlling their limbs^13,14^. A pneumatic drive is often used to reproduce the movements of a manipulator robot with a complex trajectory of movement of its parts. In this case, a combination of intelligent automatic control systems that can organize a delicate interaction between a robot and a person is effective^15^. For example, they are used in high-pressure pneumatic servo valves^16^, in the control of a throttle check valve used in an innovative two-pneumatic actuator control system^17^, industrial pneumatic grippers^18^. Industrial grippers are generally either electric or pneumatic, with the latter being preferred as their air-based functioning guarantees many advantages such as cost effectiveness and reduced encumbrance. Despite the very large employment, pneumatic grippers do not yet offer performance beyond the open/closed behavior in the majority of cases. Pneumatic grippers mounted on industrial robotic arms are commonly rigid as high forces might be required during the grasping operations^18^. Pneumatic soft robots have become the premier soft robot type among the plethora of soft robot testbeds under research, being the continuum pneumatic soft robot (cPSR) the most compliant, yet challenging due to its hyperelastic behavior of its constitutive elastomer material. In this type of cPSR, embedded pneumatic chambers materialize the pneumatic controller from within, thus, there arises two highly intertwined dynamical systems: the kinetic one due to the cPSR inertia and to the pneumatic system itself^19^. Collaborative robots are expected to physically interact with humans in daily living and the workplace, including industrial and healthcare settings. A key related enabling technology is tactile sensing, which currently requires addressing the outstanding scientific challenge to simultaneously detect contact location and intensity by means of soft conformable artificial skins adapting over large areas to the complex curved geometries of robot embodiments^20,21^. Many robotic tasks require knowledge of the exact 3D robot geometry. However, this remains extremely challenging in soft robotics because of the infinite degrees of freedom of soft bodies deriving from their continuum characteristics. Previous studies have achieved only low proprioceptive geometry resolution (PGR), thus suffering from loss of geometric details (for example, local deformation and surface information) and limited applicability^22^. Soft manipulators can perform continuous operations due to their inherent compliance and dexterity, thus enabling safe interactions and smooth movements in confined environments. However, high compliance usually means low load capacity. It is important for a soft manipulator to possess proper flexibility while maintaining an acceptable stiffness to widen its applications^23,24^. In this paper, it is shown that based on an analysis of the implemented functions of existing robot manipulators the task of automation of the safe capture of objects by a robot during the assembly process is poorly developed. In the process of analysis, there were discovered technological solutions to three main tasks for the development of a subsystem for capturing objects by a robotic manipulator: determination of the dimensions and shape of the capturing object; determination of the distance from the robot manipulator to all the points of the capturing object, determination of the capture point of the object and clarification of the distance to the robot manipulator. It is shown that all of the above tasks are not sufficiently solved^25,26^.

The results of the evaluation show that the practice in laboratories contributes to the improvement of the necessary technical abilities of students^27,28^. Practical experimentation, which combines production processes and advanced learning technologies, is important for future academic and applied engineering staff. One of the advantages of a highly qualified engineer is the ability to debug mechatronic systems, and detect and diagnose faults. For this purpose, special training laboratory stands^29^ and interactive electropneumatic and electrohydraulic simulators^30^ are being developed, and these technologies have also been used in the development of fault-tolerant mechatronic systems.

Due to the extraordinary circumstances caused by COVID-19, and from February 24, 2022, and the martial law in Ukraine, various alternatives to face-to-face classes, online platforms for learning practical work, and modeling of mechatronic systems^31^, such as Autodesks, are becoming increasingly popular. Tinkercad (for Arduino programming and modeling), Factory I/O (for training and modeling of production line automation processes), FluidSIM (for studying and modeling pneumatic, electropneumatic and hydraulic systems)^32,33^. Today, there are many initiatives that contribute to the study of science, technology, mathematics, robotics and mechatronics. Although there are many options, most of them either require large economic investments or require a lot of time for the development of educational activities^34,35^. Electropneumatic simulators are being developed as an auxiliary tool for the existing typical teaching and learning process. Based on the assessment of teachers of practical work with the use of such simulators, there is an increase in student achievement to 40%^36^. In addition to the advantages of developing tools for remote learning of mechatronic systems, it is widely known to use a variety of models for preliminary research in the development of new and modernization of existing industrial automatic control systems^37,38^. Pneumatic actuators and manipulators are becoming more common in the development and implementation of pneumatic artificial muscles in bionic work^39^.

Therefore, preliminary mechatronic studies electropneumatic systems with the use of special laboratory equipment and the expansion of research capabilities by means of simulation is an urgent task for both qualified professionals and students during training.

### Description of the laboratory stand

**Figure 1:**
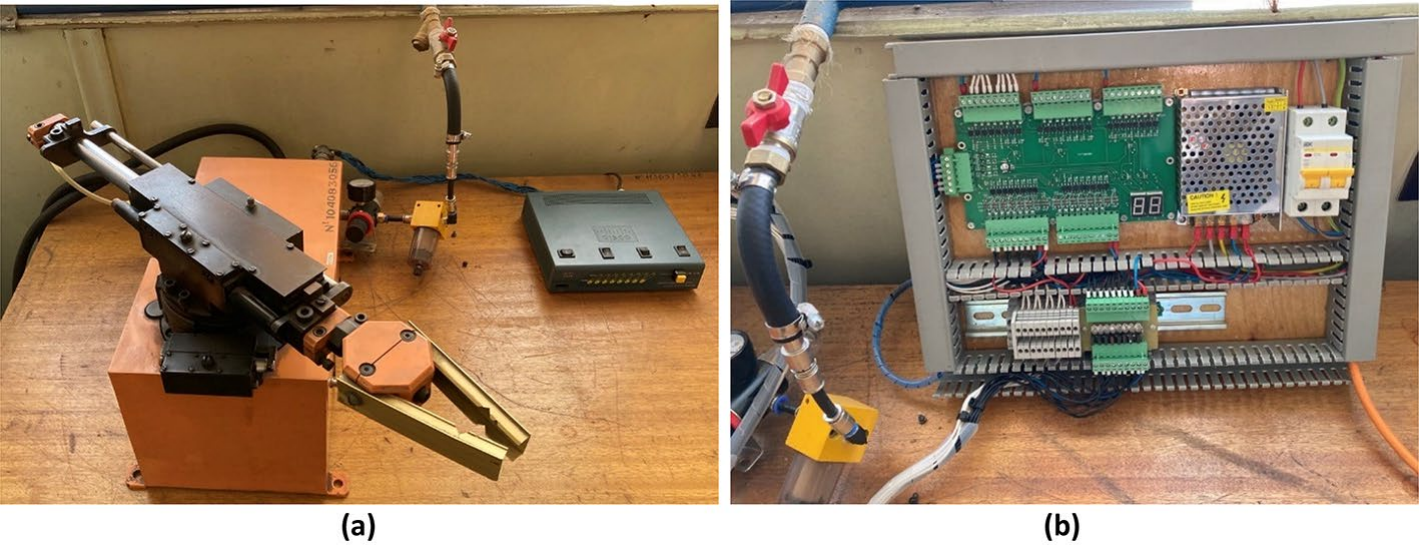
MP-9C manipulator with (**a**) manual control panel, (**b**) microcontroller part of the stand.

Mechatronics—is a branch of science and technology based on the synergistic combination of precision mechanics with electronic, electrical, and computer components that provide the design and production of qualitatively new modules, systems, and machines with intelligent control of their functional movements^3,40–42^. Modernization of the obsolete ACS pneumatic manipulator MP-9C allows to the development and consolidation of all the necessary skills in the field of mechatronics due to the presence of pneumatic, electrical, and microcontroller subsystems, and two control options (manual/software) will analyze the quality of control in both modes. For the operation of the manipulator in a manual mode, the control panel where each switch corresponds to one direction of movement of the captor is developed (Fig. 1a).

The industrial robot MP-9S is designed to perform transportation and orientation of parts. The robot can work as part of robotic systems and flexible automated production. The drives of all links and the gripper of the robot are pneumatic. The specificity of the cyclic manipulator is that it can be in a finite number of states. The MP-9S robot manipulator is a mechanism with three degrees of freedom, each of which moves with the help of two pneumatic actuators and has two positioning points. Thus, the reachable area of this manipulator is a finite set consisting of eight points.

Manipulator parameters: load capacity, including gripper mass—0.2 kg, power consumption -250 W, manipulator weight—32 kg, mass of the control device—18 kg, maximum movement speed of the manipulator links: degree of mobility 1 (vertically)—0.12 m/s, degree of mobility 2 (turn)—300 m/s, degree of mobility 3 (extension of the grip)—0.75 m/s; the maximum amount of movement of the manipulator links: degree of freedom 1—30 mm, degree of freedom 2—120 mm, degree of freedom 3—150 mm.

There are two ways to program a robot: learning and programming with some algorithmic language. The first method is simple and does not require a high qualification of a human operator, but does not allow programming complex technical processes. Language programming is more promising, since it has practically no restrictions on the level of complexity of the programs being created and allows interactive control of robots. There are two different approaches to creating a robot control language. One of them is to develop a new language specifically designed for programming robotic tasks. This approach assumes, in particular, that the syntax of the language is adapted to the description of the behavior of the robot, that is, it is as understandable and economical as possible. An alternative approach is to use traditional high-level universal programming languages for solving robotics problems, provided that the chosen language allows you to define the necessary data structures and manipulator control commands.

Compressed air is supplied to the electropneumatic valves of the manipulator through the air preparation unit, which provides regulation of the required pressure, supply of air and lubricant to pneumatic cylinders. The manipulator has electro-pneumatic valves installed for each movement. Each valve is equipped with a throttle installed at the outlet, the adjustment of which allows changing the speed of movement. The movement of the manipulator is carried out along the adjustable end stops. The sequence and number of movements, according to the accepted technological scheme, is carried out by the set of the program on the remote control. The signal about the execution of each movement is given by sensors when the permanent magnets installed on the moving parts approach them. Only after receiving the response signal about the execution of the movement (command), the command for the execution of the next movement is issued.

In the absence of a signal from the sensor to perform a movement, according to the program, the manipulator stops and no further movements occur until the signal is received. Damping of extension and rotation of the manipulator arm is carried out by hydraulic dampers. Amortization of the raising (lowering) of the arm is carried out by throttling the supply and removal of air.

The compressor provides air supply. Air preparation unit—regulates pressure, air supply, and lubrication in pneumatic cylinders. An electropneumatic valve with a throttle is installed in the manipulator for each movement to change the speed of movement.

To implement the software mode of control of the manipulator mechatronic system, a microcontroller part of the stand based on ADuC841 was developed (Fig. 1b), in which it is possible to set a certain sequence of signals that simulates part of the technological process.

Varying the number of degrees of freedom for the manipulator, allows you to diversify the simulated processes. End position sensors allow you to use the feedback for the correct operation of the program, and accordingly the manipulator, and the execution of the required sequence of commands. In the absence of a signal from a certain sensor, the following action cannot be performed, this is a certain way to reduce unexpected movements that can lead to injuries to students during classes.

### Computer modeling of mechatronic system of electropneumatic manipulator and control systems in FluidSIM

**Figure 2:**
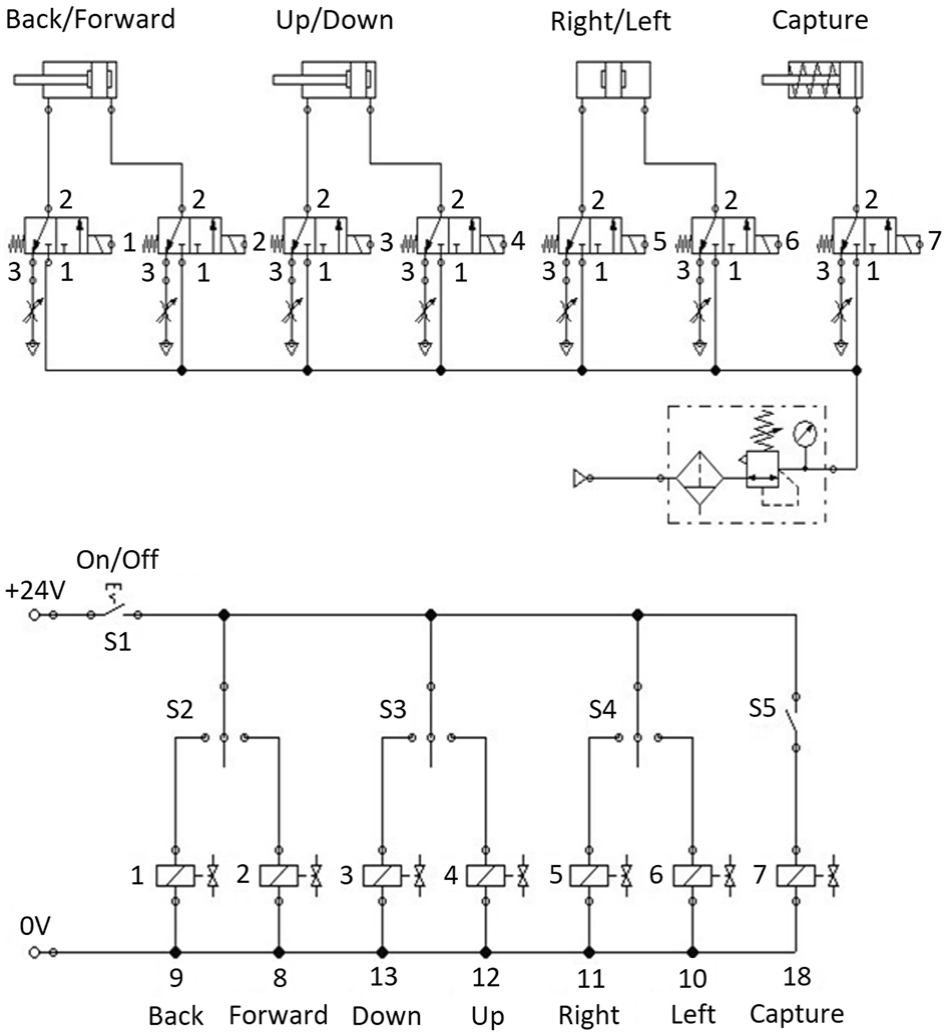
Computer model of the mechatronic system of electropneumatic manipulator.

The computer model of the mechatronic system of the electropneumatic manipulator is developed in the software package FluidSIM (Fig. 2), which is widely used for the study and research of electropneumatic and electrohydraulic systems, has didactic properties such as package modularity and user-friendly graphical user interface different methods of modeling and control.

When the S1 button is pressed, power is supplied to the manipulator. Switch S2 is responsible for the movement of the manipulator's arm forward/backward, S3—up/down, S4—turn left/right. The S5 button is responsible for the operation of the catcher. When changing the position of a particular switch, power is supplied to the appropriate electropneumatic distributor. This changes its position, thereby changing the direction of air movement. Thus, the cylinder rod is extended or retracted. Lowering, raising, and turning the arm of the manipulator is performed using double-acting pneumatic cylinders. The hand of the manipulator is designed to push the gripper into the working area, where the capture, movement, and installation of the part. The return stroke of the gripper piston is provided by a return spring.

**Figure 3:**
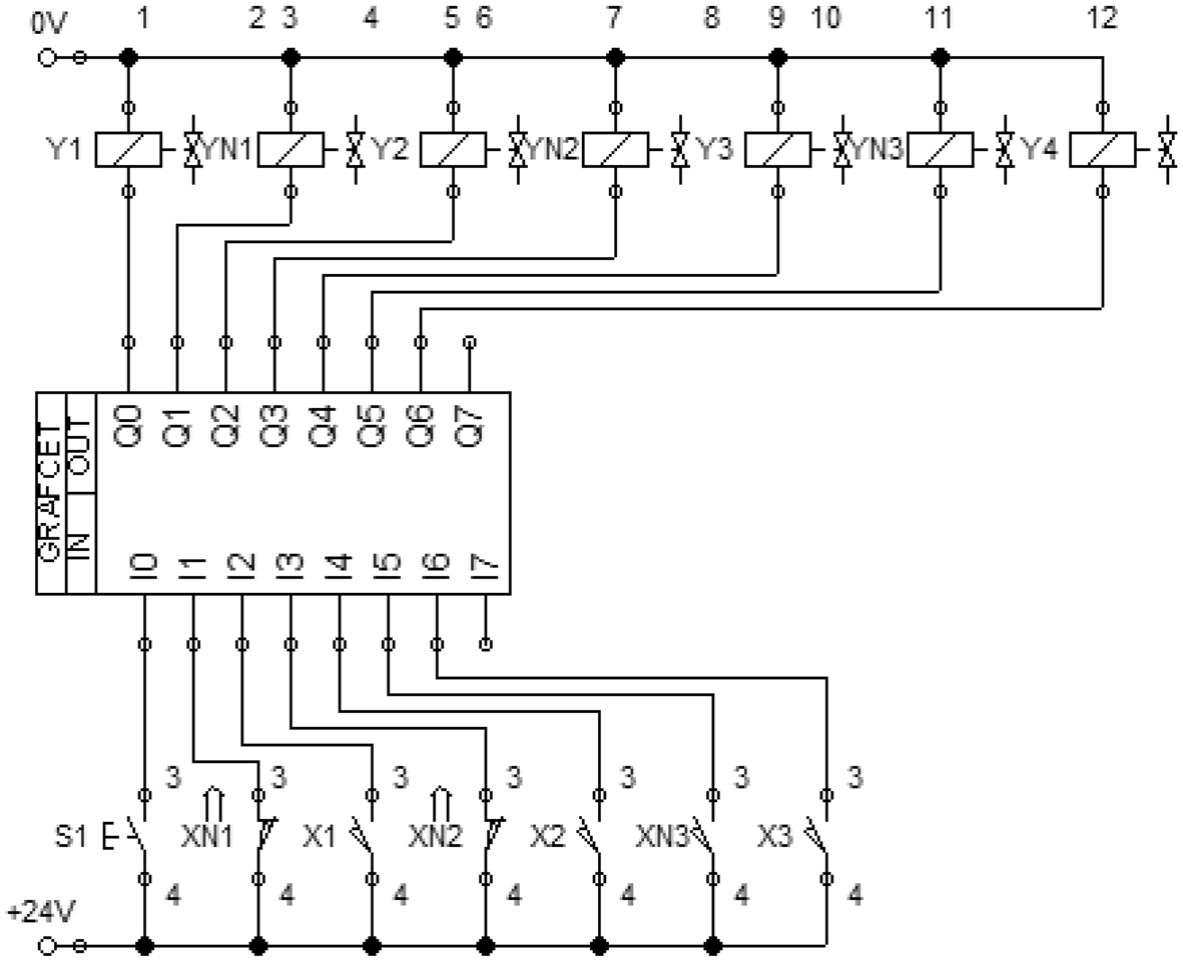
Computer model of electropneumatic manipulator control system based on GrafCet technology.

FluidSIM software provides various advantages in the process of developing and designing control systems, such as system visualization in the design and tracking of real-time simulation parameters^16^. In the control circuit, the shortness of the signals does not always allow to solve the problem of testing the required cyclogram drives, so there is a need to use additional distributors that convert the pulse signal into a permanent one, i.e. "remember" to a special signal that resets. To avoid these difficulties, you can use GrafCet technology, which provides step-by-step execution of the entire cycle^15^. Therefore, based on GrafCet technology, the automatic mode is modeled (Fig. 3).

**Figure 4:**
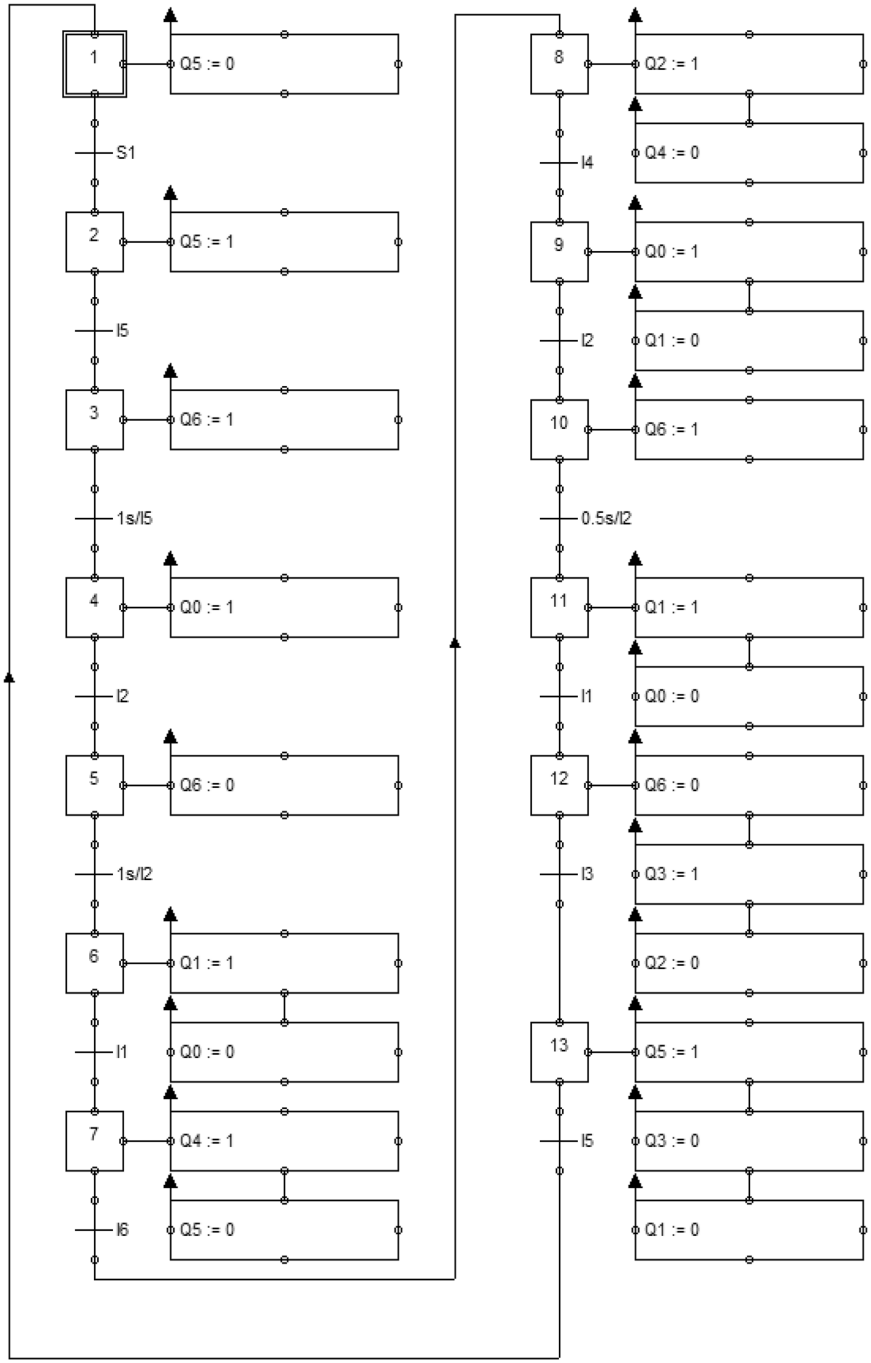
GrafCet algorithm.

Figure 4 shows the GrafCet algorithm with the following steps: 1. Waiting to confirm the start of the cycle; 2. Rotate the manipulator to the first position; 3. Unlocking the gripper; 4. Nomination of the gripper; 5. Capture the workpiece; 6. Return the gripper to its original position; 7. Rotate the manipulator to the second position; 8. Lifting the manipulator; 9. Nomination of the gripper; 10. Unlocking the gripper; 11. Return the gripper to its original position; 12. Lowering the manipulator and locking the gripper; 13. Rotate the manipulator to the first position.

This diagram can be a specific instruction for future debugging of a real object. The scheme of the sequence of technological operations makes it easy to identify inconsistencies in the work, because each stage is a separate step with a unique number.

Currently, in the development of control programs on industrial controllers, pre-simulation is widely used. This allows you to develop a program, test its performance, and efficiency and reduce the risk of damage to real equipment during debugging.

FluidSIM environment can act as a tester for a real industrial controller. Thanks to the intuitive interface, the user quickly masters the stages of connecting a virtual controller with real code to FluidSIM, and with the help of simulation mode or even GrafCet, it is easy to check the operating modes of the industrial microcontroller and make some corrections if necessary. You can download the customized program to real equipment and use it. In this way, pre-testing in the FluidSIM environment minimizes code errors and prevents severe industrial accidents.

**Figure 5:**
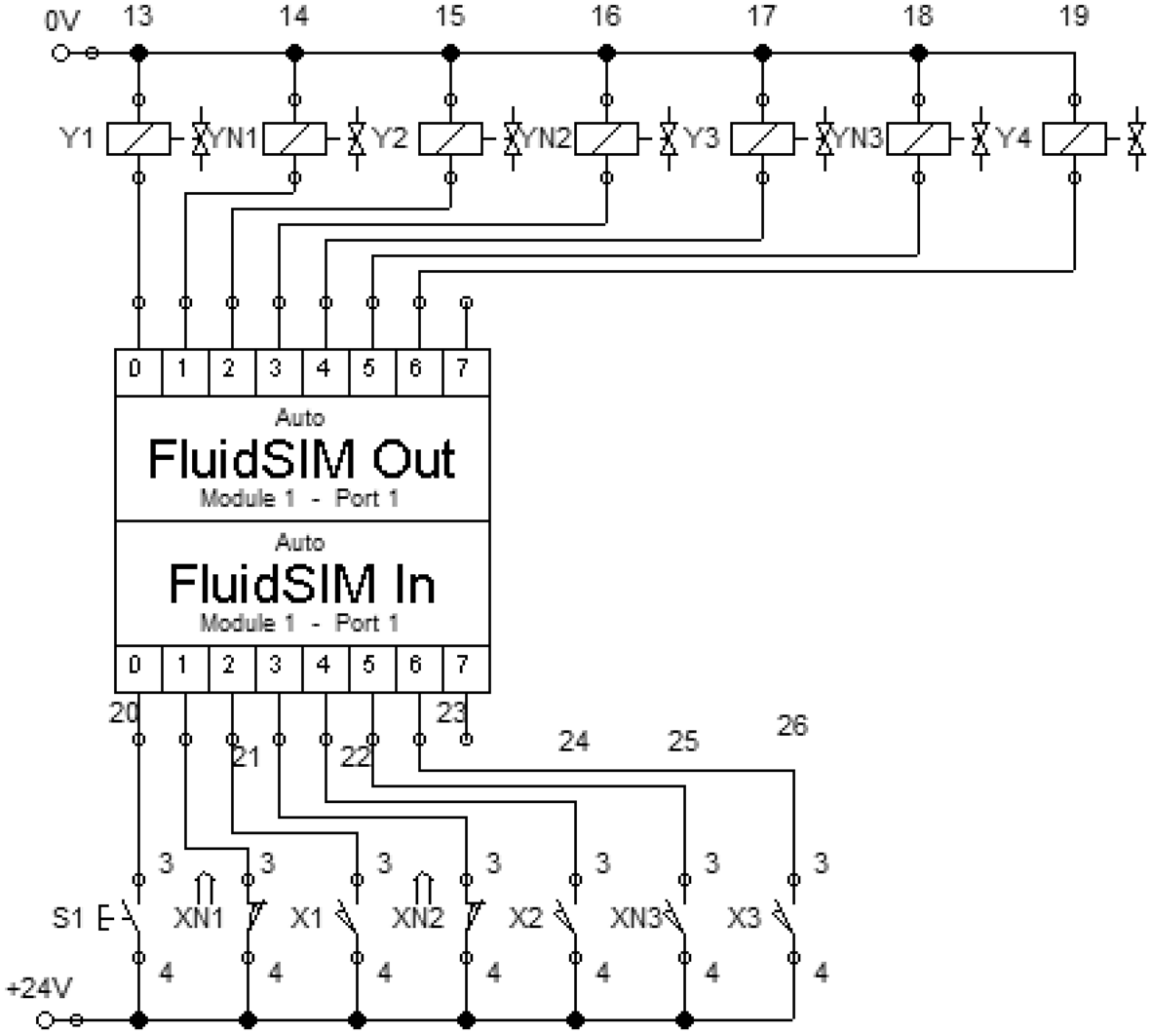
Control system with units for connection to virtual controllers.

An example of connecting a Siemens virtual controller S7-1200 to the model of the manipulator in the FluidSIM environment is presented in Fig. 5. That is, using the software environment STEP-7 (or TIA-Portal) the control program for the real industrial Siemens controller is developed. Festo EzOPC software transfers information from STEP-7 to FluidSIM and the manipulator model is controlled. Therefore, when connecting real equipment with an already established controller program (due to simulation), the risk of equipment damage is reduced. Using this method of testing is useful both for teaching students and for use in the workplace.

**Figure 6:**
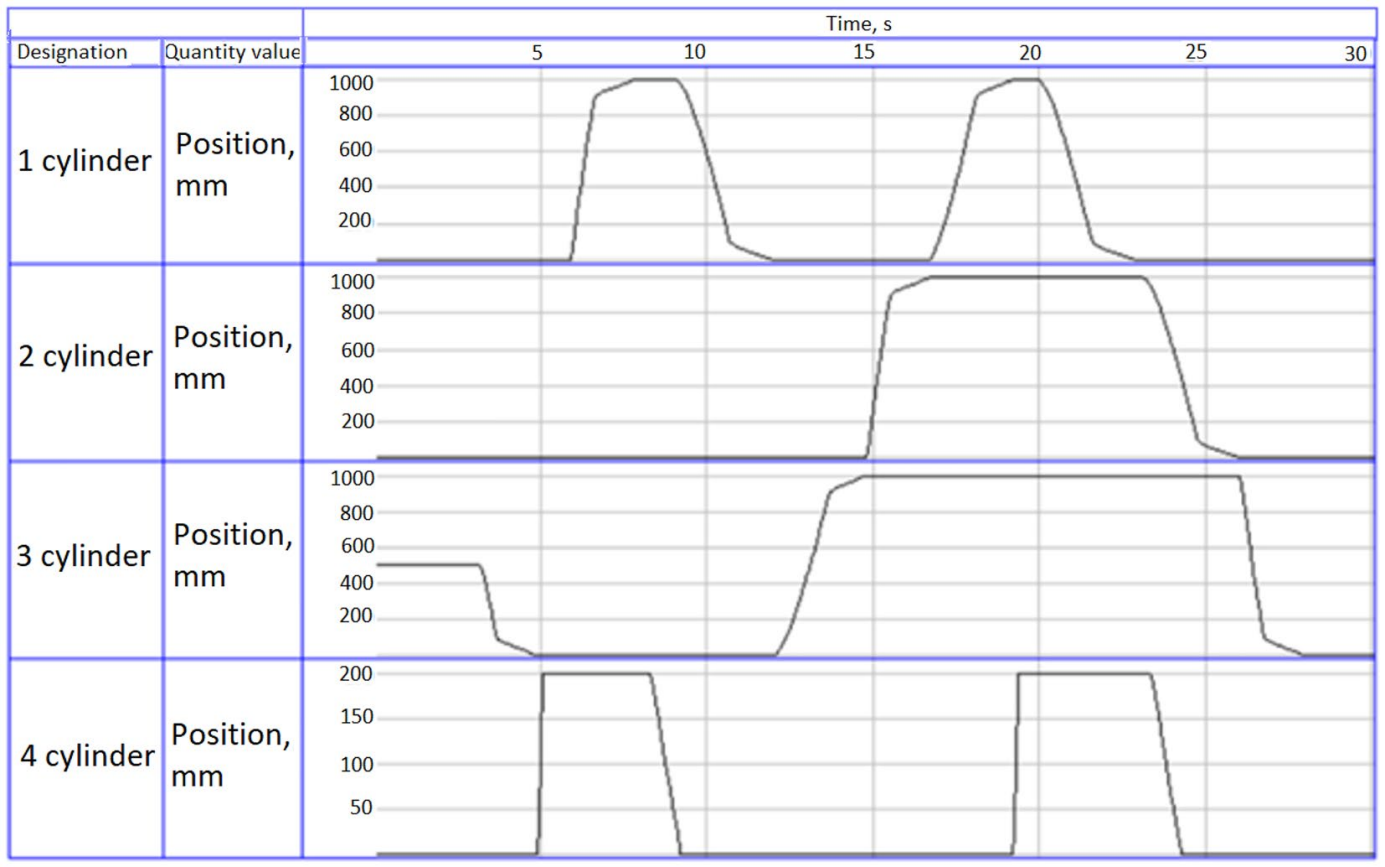
Graphs of movement of cylinders of the electropneumatic manipulator.

The result of the program is presented in Fig. 6. On the diagrams, you can visually trace the sequence of operations that correspond to the algorithm in Fig. 5. Cylinder 1—controls the rotation of the manipulator to the left/right; cylinder 2—movement of the grabber forward/backward; cylinder 3—controls the capture of the workpiece; cylinder 4—movement of the manipulator up/down.

The deceleration at the end of the movement of each of the first three cylinders is due to the presence of damping. The fourth cylinder is a spring-loaded grip, without damping.

Thus, mastering the skills of interconnecting programs and mastering the structure of real industrial controllers during classroom classes, allow students to adapt more quickly in production and improve its efficiency.

The best indicator of the efficiency of the automatic cycle before the manual is the speed of operations, accuracy, and minimization of errors in the sequences of movement. In order to compare the manual and automatic modes, physical experiments were carried out on the stand during which a video recording of the movement of the manipulator elements was made. As a result of video processing, the following results were obtained (in parentheses are the moments of time for manual and automatic modes, respectively): the beginning of opening the grip (0.00/0.00 s); the grip is open (0.14/0.12 s); beginning of advancement (1.22/0.54 s); end of extension (2.66/2.00 s); beginning of closing grip (3.70/2.36 s); the grip holds the object (4.08/2.76 s); the beginning of the rise (4.96/3.22 s); end of rise (5.20/3.44 s); beginning of retraction (6.22/4.08 s); end of retraction (7.70/5.60 s); the beginning of the turn (8.46/5.82 s); end of turn (8.94/6.32 s); the beginning of the opening of the rapture (9.90/6.46 s); the grip is open (10.02/6.60 s); the beginning of the turn with the simultaneous closing of the grip (10.88/7.22 s); end of turn (11.30/7.62 s); beginning of descent (12.28/7.78 s); end of descent (12.50/8.02 s).Thus, the results of the study showed that in manual mode, the average cycle time was 12,5 s, which is 1.5 times longer than the automatic cycle.

Transient processes obtained with the help of a laboratory bench, describing the positioning of four interconnected electropneumatic cylinders of the manipulator, have a control error that does not change its sign and the output value gradually approaches the set value. The graphs have sloping sections with a duration of about 1 s, which worsen the quality indicators of the positioning control system, namely the time constant of the transition process.

In the future, it is planned to use the developed mechatronic system of the electropneumatic manipulator for giving of preparations on the conveyor of sorting station, and also for research of indicators of quality and adjustment of system of automatic control. It is planned to continue further research in the direction of finding the optimal mode of operation of the pneumatic manipulator from the point of view of the ratio of speed and consumption of compressed air, which will allow to increase the energy efficiency of similar industrial mechatronic systems.

## Conclusions

The developed mechatronic automatic system of the electropneumatic manipulator allows investigating of control algorithms in the manual and automatic mode which can be realized by relay-contactor means, on the basis of GrafCet technology and with the use of the virtual controller.

The innovativeness of the developed mechatronic automatic system of the electropneumatic manipulator implies the variability of the control algorithms, the quality of which can be investigated on the same control object. It represents an innovative engineering solution that can be used for education and industry.

The use of a laboratory stand with an adequate simulation model, which is a comprehensive tool for developing, debugging, and evaluating the effectiveness of control systems for the electropneumatic manipulator, reduces the complexity of developing control systems for practical applications.

The combination of computer modeling of technological processes and physical modeling of executive mechanisms increases the accuracy of reproduction of real equipment physical processes, which allows to reduce material costs for conducting experiments on real equipment.

The transient processes of the mechatronic system of automatic control of the pneumatic manipulator obtained as a result of the study in automatic mode allow the full list of manipulator functions to be performed 1.5 times faster than in manual mode. Transient processes obtained with the help of a laboratory bench, describing the positioning of four interconnected electropneumatic cylinders of the manipulator, have a control error that does not change its sign and the output value gradually approaches the set value. The graphs have sloping sections with a duration of about 1 s, which worsen the quality indicators of the positioning control system, namely the time constant of the transition process.

It is planned to continue further research in the direction of finding the optimal mode of operation of the pneumatic manipulator from the point of view of the ratio of speed and consumption of compressed air, which will allow to increase the energy efficiency of similar industrial mechatronic systems.

Mechatronic systems are used to automate and optimize processes on industrial production lines. They make it possible to improve production processes, ensuring high accuracy and efficiency. The study of pneumatic manipulators will improve efficiency and productivity, optimize their speed and accuracy for various applications in production. Research on the interaction of pneumatic manipulators with other technologies, such as machine learning, artificial intelligence, IoT is the basis for creating more integrated and intelligent systems.

## Data Availability

Abstracted data is available from the corresponding autor on reasonable request.
